# 45,X/46,XY Mosaicism and Normozoospermia in a Patient with Male Phenotype

**DOI:** 10.1155/2019/2529080

**Published:** 2019-01-20

**Authors:** Shathmigha Ketheeswaran, Birgit Alsbjerg, Preben Christensen, Claus Højbjerg Gravholt, Peter Humaidan

**Affiliations:** ^1^The Fertility Clinic Skive Regional Hospital, Skive Regional Hospital, Resenvej 25, 7800 Skive, Denmark; ^2^Faculty of Health, Aarhus University, Aarhus, Denmark; ^3^SPZ Lab A/S, Fruebjergvej 3, 2100 Copenhagen, Denmark; ^4^Department of Endocrinology and Internal Medicine, Aarhus University Hospital, Aarhus, Denmark; ^5^Department of Molecular Medicine (MOMA), Aarhus University Hospital, Aarhus, Denmark

## Abstract

The phenotypic spectrum of 45,X/46,XY mosaic males varies greatly. Previous reports have only described cases with either oligozoospermia, growth retardation, or elevated gonadotropins. However, the present case presented with normozoospermia, and normal height, sperm DNA fragmentation index (DFI), and gonadotropins. The male and his spouse were referred to The Fertility Clinic, Skive Regional Hospital, due to 2 years of infertility. After failure of several attempts of assisted reproductive treatment (ART), the male underwent genetic analysis. Conventional karyotyping in peripheral lymphocytes yielded a low-grade 45,X/46,XY mosaicism, confirmed by fluorescence in situ hybridization (FISH) showing 6% 45,X cells. A FISH test performed on interphase nuclei from buccal mucosal cells yielded one cell with only one X-signal (0.6%), explaining the normal phenotype of the patient, but not the infertility. FISH test for sperm aneuploidy showed normal range parameters, except for a 10-fold elevated gonosomal nullisomy rate (2.1%). Hence, germinal mosaicism may be an explanation of the infertility of the case. Increased sex nullisomy levels may reflect an aberrant testicular environment compromising fertility even though sperm euploidy rates and other sperm parameters do not preclude a successful treatment with ART. Based on these results, the couple decided to use donor semen for their subsequent intrauterine insemination treatment and obtained a successful pregnancy.

## 1. Introduction

Sex chromosome 45,X/46,XY mosaicism is characterized by the expression of both cell lines within an individual [1]. The genotype results in a broad phenotypic spectrum, ranging from females with or without Turner-like stigmata, to males and females with gonadal dysgenesis and to phenotypical normal males [1–4]. There is no correlation between the proportion of mosaicism in peripheral blood cells i.e., cultivated lymphocytes and the clinical expression [1, 4, 5]. This applies for mosaic males with structural abnormalities of the Y chromosome as well [3].

The incidence of sex chromosomal mosaicism, including 45,X/46,XY, is estimated to be 1.5 of 10,000 [6] to 1.7 of 10,000 [7], thus indicating that it is a rare condition. Female phenotypes are overrepresented [8] as they may be diagnosed due to a host of causes whereas males are only diagnosed when encountering infertility complications during adulthood, or growth retardation or genital abnormalities during childhood [1, 2]. In addition, increased risk of developing cardiovascular diseases and cancer among 45,X/46,YX mosaic karyotypes have been reported [9, 10]. To date, case reports or series [1–4, 6, 9, 11, 12] and larger studies [8, 13, 14] regarding 45,X/46,XY mosaic males have described growth retardation, elevated gonadotropins, azoospermia or severe oligozoospermia, and high sperm DNA fragmentation index (DFI). We herein present a case with 45,X/46,XY mosaicism and infertility despite normal semen parameters and normal DFI.

## 2. Case Report

A 39-year-old male was referred to The Fertility Clinic, Skive Regional Hospital, Denmark in 2013 along with his spouse due to primary infertility. They had attempted pregnancy for two years and throughout the treatment period the male delivered normal semen specimens according to 2010 World Health Organization (WHO) criteria [15] (Table 1). Furthermore, gonadotropin and sex hormone levels were normal (Table 1) [16–18]. A test for sperm DNA integrity (SDI-test) showed only a small proportion of damaged sperm cell DNA as DFI was 9.1% (normal range below 15%).

Regarding the clinical examination of the genitals, no abnormality was found. Both testes were of normal size (20 and 15 mL, respectively); the male had normal virilization and normal development of the penis, and ultrasound examination of the testes showed no abnormalities. Furthermore, the medical history of the male was normal with no recorded events affecting spermatogenesis and no familiar disposition to fertility disorders or other conditions. His height was 181 cm, weight was 71.9 kg, and BMI was 21.9 kg/cm^2^. In addition, an echocardiography showed a normally structured heart without coarctation of the aorta. A standard chromosome analysis based on 10 metaphases from cultivated peripheral lymphocytes in Q-band yielded a low-grade 45,X/46,XY mosaicism. Here, 1 out 10 metaphases contained a 45,X cell line while the remaining 9 contained 46,XY. This result was confirmed by a second karyotype, using fluorescence in situ hybridization (FISH) analysis, in peripheral lymphocytes screening 100 metaphase lymphocytes at 400–450 band resolution with specific probes for chromosome X. Out of 100 metaphases, 6 presented 45,X karyotype, while the remaining 94 presented regular 46,XY karyotype. In order to test for confined tissue mosaicism, FISH analysis, with probes for chromosomes 18, X and Y in mucosal cells from a buccal swab, was performed. The analysis of 162 interphase nuclei yielded one cell with only one X-signal (0.6%), thus showing no gonosomal mosaicism above the cutoff value of 3.0% [19] (see discussion).

In addition, a test for sperm aneuploidy was performed using FISH with probes for chromosomes 13, 18, 21, X and Y. The frequency of gonosomal nullisomy was markedly elevated with 2.1% of the sperm containing neither a X nor a Y chromosome. Other values for this sample were within the normal range [20, 21].

The couple underwent several ART treatments from the inception of their referral to obtaining pregnancy with donor sperm intrauterine insemination (IUI-D). Initially, the couple underwent three IUI attempts using the sperm from the male. Thereafter, they underwent two in vitro fertilization (IVF) attempts and two attempts of combined IVF and intracytoplasmic sperm injection (ICSI). During these treatments, the spouse had a total of 45 metaphase II (MII) oocytes retrieved and 14 good quality embryos were transferred during fresh and frozen thawed transfers. None of the treatments resulted in neither biochemical nor ultrasound verified viable pregnancies. The couple decided to continue treatment using donor semen and subsequently obtained an ongoing pregnancy after their third IUI-D. A statement of consent was obtained from the patient.

## 3. Materials and Methods

The male delivered a semen sample on-site after 2–5 days of ejaculatory abstinence. The semen samples were analyzed according to 2010 WHO criteria for the examination of human semen [15], and the preparation was made by gradient centrifugation (Table 1).

First karyotyping of peripheral lymphocyte was performed with a standard chromosome analysis based on 10 metaphases from cultivated peripheral lymphocytes in Q-band. Second karyotyping was performed by FISH analysis with screening of 100 metaphase lymphocytes at 400–450 band resolution with specific probes for chromosome X, and both analyses were performed at Aarhus University Hospital.

Sperm DNA integrity was investigated with the SDI-test (SPZ Lab, Denmark). Briefly, 0.5 ml semen was diluted with TNE buffer, mixed and frozen directly in liquid nitrogen. After thawing, fluorescent staining was performed according to the SCSA protocol [22]. The samples were analyzed using a FACSCalibur (BD Biosciences) flow cytometer. Data were acquired using the CellQuest software (Version 3.2., BD Biosciences). Each analysis was run in duplicates and recording was stopped after acquisition of 5000 events.

Sperm aneuploidy test was performed as described in [21]. Thus, a total of 1013 spermatozoa were analyzed by FISH with specific probes for chromosome 13, 18, 21, X and Y.

FISH analysis, in mucosa cells from a buccal smear with specific probes for chromosome 18, X and Y, was performed using an in house modification of the method described by Bartsch and Schwinger [23]. A total of 162 interphase nuclei were analyzed. Both investigations were carried out in the laboratory of gametoGen Gmbh, Hamburg, Germany.

## 4. Discussion

In this report, we present an infertile male with normal semen analysis, normal hormonal levels, and normal height who, however, showed a low-grade 45,X/46,XY mosaicism in peripheral lymphocytes. Conventional cytogenetic analysis, including FISH of 100 metaphases, yielded a 45,X/46,XY mosaic state with 6% 45,X cells. Testing a different tissue, such as buccal mucosa cells by FISH, seemed appropriate in this case, in order to differentiate between true mosaicism and mosaicism confined to blood. Former studies suggested that blood cells were of extraembryonic origin, thus explaining karyotypic discrepancies between blood and other tissues. The yolk sac was proposed as the ultimate origin of the lymphohematopoietic precursors. Subsequent studies identified a region associated with the dorsal aorta as the primary site of “definitive” stem cells. These opposing views are currently achieving a compromise that recognizes that both sites contribute stem cells involved in seeding the developing tissues [24]. A FISH test, with probes for chromosomes 18, X and Y in 162 interphase nuclei from buccal mucosal cells, yielded one cell with only one X-signal (0.6%), thus showing no gonosomal mosaic state above the cutoff value of 3.0% [19]. Notwithstanding, this result explains the unremarkable phenotype of the patient; it does not exclude the possibility of a mosaic state in the gonads of the patient. Consequently, a sperm aneuploidy test was performed using FISH analysis with probes for chromosomes 13, 18, 21, X and Y. In this sample, all parameters, but one, were within normal range. Noticeably, the percentage of XY, XX, and YY disomies in sperm cells were not elevated. However, the frequency of sperm containing neither a X nor a Y chromosome (gonosomal nullisomy) was approximately 10-fold elevated (2.1%) compared to normal controls [20]. The results do not fully verify germinal mosaicism in the patient, and thus, the condition may be a possible cause of infertility in the present case.

Reviewing the literature, 45,X/46,XY mosaicism in males usually results either in azoospermia or severe reduced sperm production, elevated gonadotropins, and short stature [3, 4, 6, 9, 12]. Layman et al. [3] presented three adult cases with 45,X/46,XY mosaicism in which one adult showed growth retardation as well as elevated gonadotropins and azoospermia. Furthermore, the second male presented with normal stature, but with azoospermia and elevated gonadotropins, and the third case, the one most similar to the described case in the present case report, had normal stature, normal serum testosterone, and normal gonadotropins. However, this case presented with severe oligozoospermia. In peripheral blood lymphocytes, the third case demonstrated 26% 45,X cells compared to the 6% 45,X cells in the present case. Interestingly, Mohammed Lashkari et al. [6] reported 49 infertile males with 45,X/46,XY mosaicism in peripheral lymphocytes of whom, four cases were normozoospermic. Nonetheless, two of them presented with varicocele and the third had a high DFI. The fourth case, similar to the present case, had high sperm counts; however, he had a rate of mosaicism of 9.5%, and hormone levels, height, and other information were not stated specifically for each male. Moreover, Newberg et al. [12] reported a 45,X/46,XY mosaic male with primary infertility and a normal phenotype who had only moderate oligoasthenoteratozoospermia. Cytogenetic analysis of somatic cells and determination, by FISH analysis, of aneuploidy frequencies for the gonosomes of this patient yielded a mosaic 45,X (10%)/46,XY (90%) karyotype. Significantly higher frequencies of gonosomal (1.92% versus 0.70%) and chromosome 18 (0.89% versus 0.28%) disomy were detected in the sperm of the patient compared with those observed in spermatozoa from a proved fertile control. Notably, increased disomy rates were reported by Calogero et al. [20] in patients with oligoasthenoteratozoospermia. Thus, the findings in the patient of Newberg et al. [12] was most probably related to the oligoasthenoteratozoospermia rather than to the karyotypic abnormality.

For reasons discussed above, there is no correlation between clinical manifestation and percentage of mosaicism in peripheral blood lymphocytes [3–5]. The unremarkable phenotype of the presented case might be explained by the normal result of the FISH test in mucosal cells. However, this finding does not explain the infertility. Interestingly, all three cases in [3], the cases in [6], the case reported by Newberg et al. [12], and the currently presented case were diagnosed with 45,X/46,XY mosaicism due to infertility.

Infertility among males and growth retardation among boys are the common reasons for phenotypical males to be diagnosed with 45,X/46,XY mosaicism; otherwise, they remain undiagnosed [3]. 90% of prenatal diagnosed 45,X/46,XY mosaicism cases present as normal boys at birth [13, 14]. In contrast, only 11–12% of postnatal diagnosed mosaic cases present with male phenotype [1, 3]. A possible explanation why males with 45,X/46,XY mosaicism are often undetected is that the manifestation of the karyotype is less obvious. Supporting the statement, Wu et al. [4] observed that short stature was more common among females than male mosaicism cases. In contrast, Lindhardt Johansen et al. [25] observed an over-representation of males compared to females.

Notwithstanding body height within normal range and normozoospermia, the fertility may be severely compromised in 45,X/46,XY mosaic males. As in the presented case, the cause of infertility is not always explained by the semen parameters. Increased rates of gonosomal disomies in sperm have been described in mosaic males. However, these were within the range observed in other patients with oligoasthenoteratozoospermia [20]. Interestingly, the sperm FISH analysis in the present patient yielded a 10-fold increase in the nullisomy rate for the X and Y chromosome as compared to normal controls [20].

The increased rate of nullisomic sperm is remarkable as this has been described in other cases of 45,X/46, XY mosaicism [26, 27] as well as in infertile males with other mosaic states of gonosomes [28]. However, it has not been found in infertile males with abnormal semen parameters and with normal karyotypes [20] with the possible exception of men with hidden mosaicism and deletions of the long arm of the Y chromosome [26]. The sex nullisomy level observed in sperm of 45,X/46,XY mosaic males was much lower than theoretically expected when assessing the percentage of 45, X cells in blood cells, indicating that the majority of the 45,X cells resulting in nullisomic spermatozoa may have been eliminated during meiosis [27].

## 5. Conclusion

In conclusion, true mosaicism can be determined by testing several types of tissue as mosaicism can be confined to blood. The latter patients may complete spermatogenesis and produce mature spermatozoa, and thus, have normal sperm parameters. However, it may be speculated that an increased sex nullisomy level seen in patients with a mosaic 45,X/46,XY germ cell composition and other abnormal sex chromosome karyotypes reflects an aberrant testicular environment compromising fertility even though sperm euploidy rates and other sperm parameters do not preclude a successful treatment by ART.

## Figures and Tables

**Table 1:** Values of semen sample and hormonal status of the patient.

Semen sample^*∗*^	Value	Lower reference limit
Total motile count (million)	40–89.0	40
Motile grade	3–4	0–4; 4 highest
Volume of ejaculate (mL)	0.8–1.5	1.5
Sperm concentration (million/mL)	33–150	15
Total concentration (million)	50–120	39
Total progressive motility (%)	40–89	32

Hormone levels	Value	Reference

FSH (IU/L)	1.3	1.2–15.8
LH (IU/L)	1.8	1.7–8.6
Testosterone (nmol/L)	10.2	10.4–32.6
Estradiol (nmol/L)	0.06	0.065–0.208

^*∗*^Semen sample values are taken after preparation.
